# Towards regional scientific integration in Africa? Evidence from co-publications

**DOI:** 10.1016/j.respol.2022.104630

**Published:** 2023-01

**Authors:** Mafini Dosso, Lorenzo Cassi, Wilfriedo Mescheba

**Affiliations:** a European Commission, Joint Research Centre, Dir. B. Growth and Innovation, Unit B.3, Urban and Territorial Development, Seville, Spain; b Paris School of Economics - University of Paris 1 Panthéon-Sorbonne, Paris, France; c HCERES-OST, Paris, France

**Keywords:** Scientific collaborations, African countries, Regional economic communities, Publications, Bibliometrics, Gravity model

## Abstract

Regional scientific integration is a critical pathway for the development of an integrated African research area and knowledge-based society. On the African continent, progress in scientific production and integration has remained limited, mostly led by a global or international agenda, and bound to a few top publishing nations. The high-level policy commitments and the accumulated policies and strategies developed and pursued under the various intertwined sub-regional economic groupings have, to date, only diversely contributed to policy alignment and coordination in the area of science, technology, and innovation (STI) across Africa. In this context, this paper provides a first and hence original assessment of the role of region-specific factors in shaping scientific collaboration on the continent. For this purpose, our study builds upon the proximity approach to analyse the determinants of scientific collaboration between African countries, using co-publications data from Thomson Reuters' Web of Science database as a proxy of such collaboration. Our results suggest that the majority of African regional economic communities (RECs) have not yet had a significant effect on scientific co-publication. Nevertheless, some important region-specific factors do seem to be at play, such as a shared ethnical language, membership in the African and Malagasy Council for Higher Education (CAMES), and the presence of a common European partner as a third partner in co-publication. Existing policies aimed at the development of an Africa-wide research area should aim to leverage existing and emerging regional excellence networks and novel coordination models to accelerate the process of scientific integration in Africa.

## Highlights

• African regional economic communities show very uneven progresses on STI policy making and coordination.
• The majority of regional scientific co-publications involve at least one author non-affiliated in Africa.
• The CAMES membership and ‘a common European partner’ shape African collaboration.
• Geographical distance, colonial heritage and common language show decreasing importance.

## 1 Introduction

African countries are still poorly represented in the global scientific production, both in absolute terms and with respect to their populations and economic importance (UNESCO, 2021). With the exceptions of Egypt and South Africa, most African countries have not yet developed strong links with the global scientific community (Adams et al., 2014). Considering the overall structure of the scientific collaborations network, one of the main weaknesses of African countries is their low density of regional connections, with only a few African countries strongly connected to the global scientific community; yet even these are hardly able to act as regional hubs. One consequence of this is that the whole continent seemingly occupies a peripheral position in the global scientific arena, even in the fields in which science excellence exists or is emerging, such as in agriculture and tropical medicine.

Nonetheless, recent dynamics around scientific cooperation and science excellence are raising some hopes for future improvements in the local science systems in Africa (UNESCO, 2021; Guns and Wang, 2017; World Bank, 2014). Indeed, the number of scientific collaborations between African countries is increasing, although these still represent only a small share of the total African co-publications. This change has come about with the emergence of a denser continental network of scientific collaborations over the last two decades. In parallel, a plurality of international, multi-country, and regional policy initiatives have been undertaken to strengthen continental integration on key socio-economic and trade dimensions and are now bearing some fruit. In this policy context, eight sub-regional groupings have emerged and been recognised by the African Union (AU) as Regional Economic Communities (RECs), and these are expected to be the testing grounds and pioneers for broader continental integration. Indeed, the RECs should facilitate greater regional collaboration, coordination, and the execution of regional programmes (African Union Commission, AUC, 2015). As building blocks of the African Economic Community (AEC),^1^ the RECs should also support the harmonisation of national policies in the fields of agriculture, industry, energy, education, and science and technology, among others. The present study is an attempt to assess the roles of regional factors and the RECs in shaping the process of scientific integration in Africa.

From a policy perspective, the process of integration will ultimately lead to a process of convergence among countries and a higher degree of cohesion in the whole structure. To evaluate the validity of this statement, we aim to examine the main determinants of intra-Africa collaboration and, in doing so, to assess the role of various institutions in facilitating regional collaboration towards continental scientific integration. Our analysis provides a first assessment, albeit indirect, of the process of promoting the integration of African regional science. More precisely, we investigate the determinants of dyadic collaboration at a country level and the potential integrative role played by the RECs in promoting science collaboration. In doing so, our study also discusses the influence of historical ties on the evolution of African scientific collaboration.

Following prior studies (Hoekman et al., 2010; Hu et al., 2020), this analysis employs bibliometric data on co-publications as a proxy for scientific collaboration (see Katz and Martin, 1997, for a critical assessment of this proxy). Although they represent only a small share of African co-publications (8.4 %, or 30,824 out of 368,516 co-publications), the number of co-publications with at least two African co-authors showed a 13-fold increase between 2002 and 2019, which is twice more than the increase observed in the number of co-publications involving at least one African researcher. This noticeable improvement is also visible in terms of the number of participating countries: only 19 % of possible bilateral collaborations could be observed at the beginning of the period, while 70 % of possible collaborations had occurred by the end of the period.

Our empirical analysis takes inspiration from the proximity analytical framework, which allows distinguishing between geographical, social, cognitive, organisational, and institutional proximities. A key highlight of proximity studies is that geographical proximity is neither necessary nor sufficient to explain knowledge interactions between actors (Boschma, 2005; Frenken et al., 2009; Carrincazeaux and Coris, 2011). Accordingly, our study investigates the importance of different proximity dimensions for the development of African science collaboration. We attempt to shed light on the importance of institutional proximity in participation in science collaboration as captured through common membership in a REC and in other historical regional institutions.

The remainder of this paper is organised as follows. Section 2 briefly describes the policy background of continental and regional scientific integration in Africa. It also highlights the determinants of African scientific collaboration put forward by the literature. Finally, the international co-publications involving African regional economic communities are described. Section 3 briefly lays out the rationale and relevance of the proximity framework for determining the factors behind the existing scientific collaborations. It also describes the econometric approach taken in the present study as well as the dataset and variables. Section 4 discusses the main results, with a focus on the importance of the regional factors. Section 5 concludes the paper.

## 2 Policy background of continental and regional scientific integration in Africa

### 2.1 The construction of continental and regional institutions for STI integration in Africa: an overview of activity and the uneven progress across RECs

Since the Lagos Plan of Action (LPA) for the Economic Development of Africa, 1980–2000 (OUA, 1980), the African continent has been the host to a multitude of international policy initiatives and programmes aimed at strengthening national science and technology (S&T) systems. Yet, most African countries still lag behind in the majority of science disciplines (UNESCO, 2021). And this remains the case despite it being >40 years since the LPA recognised the prominent role of autonomous and collaborative S&T and the need to invest at least 1 % of GDP to raise the level of science, technology, and innovation (STI) in Africa. The LPA also envisioned a key role for the sub-regional groupings in the overall continental integration.

Further, the continental institutional architecture for STI was extended through the creation of an African Union Development Agency (AUDA-NEPAD, previously NEPAD [New Partnership for Africa's Development]), a dedicated African Union Commission (AUC) S&T department, and the establishment of the African Ministerial Council on Science and Technology (AMCOST) in the early 2000s. Under AMCOST, Africa's Science and Technology Consolidated Plan of Action (CPA) was endorsed in 2006, with the aim to trigger further continental collaboration and the development of scientific excellence networks (NEPAD, 2006). Important challenges nevertheless remain, such as the overreliance on external support, the limited impact, and the scope of human and sustainable development (AUC, 2014; Teferra, 2013). Building on the lessons from the CPA, the Science, Technology, and Innovation Strategy for Africa (STISA-2024) was developed and provides a new policy framework to address the goals of the AU's Agenda 2063^2^ (see AU, 2019, for STISA's progress report for the period 2014–2019). In parallel, the African Academy of Sciences, AUDA-NEPAD, and international partners set up the pan-African platform the Alliance for Accelerating Excellence in Science in Africa (AESA).^3^ Noteworthily, the core implementation role of the RECs was made explicit in STISA-2024 in relation with the alignment of STI plans and the pooling and sharing of resources. In addition to the central role of the RECs for the strategy, the new continental agenda for STI recalls the technical role of other sub-regional organisations, such as the African and Malagasy Council for Higher Education (*Conseil Africain et Malgache pour L'Enseignement Supérieur* in French, henceforth the CAMES), established during the first years of independences.^4^ As an intergovernmental organisation, the CAMES was set up with the aim to enhance the coordination and harmonisation of higher education systems across the French-speaking countries on the continent. During the last few decades, the multiplication of intergovernmental or sub-regional organisations, often supported by internationally funded programmes, has gradually shaped the system into its current form. Besides the CAMES, other initiatives for university cooperation were launched in the 1960s, such as the Francophone University Agency, created in 1961 (AUF since 1998, previously called the Association of fully or partially French-speaking universities), and the Association of African Universities (AAU), which was created in 1967. Both in terms of funding and organisational development, most African higher education and research systems have been shaped by their colonial ties and they have remained vastly dependent on Europe-based and US-based organisations and foundations (Cissé, 2018; Teferra, 2013; Makosso, 2006). Yet, critical gaps remain in African's higher education systems, especially in terms of knowledge production, quality, infrastructure, and capabilities (United Nations Educational, 2015, United Nations Educational, 2021; World Bank, 2014, Materu, 2008; and Cissé, 2018, for a critical perspective on the 50 years of existence of the CAMES).

At the level of the RECs, it can be said that very few of them have taken bold steps to hasten the process of integration, including advancing STI policy coordination. There are eight RECs, as legal groupings of African States, that have been recognised by the African Union, and these comprise: the Arab Maghreb Union (AMU or UMA); the Common Market for Eastern and Southern Africa (COMESA); the Community of Sahel-Saharan States (CEN-SAD); the East African Community (EAC); the Economic Community of Central African States (ECCAS); the Economic Community of West African States (ECOWAS); the Intergovernmental Authority on Development (IGAD); and the Southern Africa Development Community (SADC). Beyond their great size heterogeneity, the constitutive and legal components of the RECs widely differ in terms of capacity development (NEPAD, 2015), citizens' rights and benefits, rules of origin, criteria for preferential treatment, product coverage for trade liberalisation, their operations, and the funding of their organs, as well as in terms of the rules of power and the functioning models of their organs (Mangeni and Juma, 2019). Although they should pave the way for greater continental economic integration, their impacts to date have been hindered by them overlapping in certain areas, the different progress on integration, and some fragmentation due to their competing interests (Byiers et al., 2019; Tavares and Tang, 2011). Nevertheless, at the STI policy level, some improvements can be observed in several member states that have committed to the development or revising of STI strategies; this trend has also come about with initiatives launched by a few RECs to foster scientific integration (United Nations Educational, 2015, United Nations Educational, 2021). Although sub-regional policy progress remains somewhat uneven, the RECs can provide ‘fertile ground for trial and error and for learning valuable lessons’ that could be useful for broader continental integration (Mangeni and Juma, 2019, p. 59).

Our paper acknowledges these perspectives and contributes to the literature by questioning the integrative role of the RECs in promoting scientific collaboration in Africa; it does so by acknowledging that the RECs should lead the way towards broader continental integration. Table 1 below provides some key legal information on the RECs, including their respective year of establishment or adoption, website, founding treaty or agreement, secretariat headquarters, frequency or latest Summit of the Heads of State and Government, and the existing STI strategies or protocols. As the policymaking institution of regional communities, the summit takes the lead for the overall policy direction and ensures control over the functions of the RECs. Reasonably, it can be assumed that the RECs also represent the high-level policy coordination needed to advance regional integration towards an African research area. The last column in Table 1 gives the STI coordination structure of the RECs or any transversal STI organisation or institution (when the former one does not exist or is not documented). The information was mainly extracted from the AU website (AU, 2019), and cross-checked with information on the official REC website (where available) or that of the relevant thematic international organisations, such as UNESCO and AUDA-NEPAD (see Appendix B for the different sources). Table 1 illustrates the heterogeneity across the communities, including with respect to the coordination structures for STI policy. Here, EAC, ECOWAS, and SADC seem to be the most advanced communities in terms of STI policy integration (AU, 2019; Mugabe, 2011).

**Table 1:** Regional Economic Communities (RECs): legal basis (foundation) and STI policy and coordination structure.

Regional Economic Communities (number of member states)	Year of establishment and/or entry into force and treaty or agreement (city)	Secretariat's headquarters (active website, May 2021)	Head of states or governments summits or assembly (frequence and/or a recent one)	Common STI protocol or policy adopted (current)	STI coordination structure (OR other STI transversal organisation/institution)
Arab Maghreb Union, AMU (5)	1989 Marrakech Treaty	Rabat, Morocco (https://maghrebarabe.org/fr/)	Very infrequent (>10 years) also in relation to strong political divisions	No	University of Maghreb (not effective; in creation)
Community of Sahel-Saharan States CEN-SAD (29)	1998 in Tripoli (becomes a REC in 2000)	N'Djamena, Chad since 2019 (not accessible)	Summit in 2013 and then a one-day Extraordinary session in 2019	No	Not available
Common Market for Eastern and Southern Africa COMESA (19)	1993/1994 COMESA Treaty in Kampala	Lusaka, Zambia (https://www.comesa.int)	20th Summit of the Authority in 2018	No	Innovation Council established in 2012 to support the organisation of scientists/engineers, and innovation
East African Community EAC (5)	1999/2000 EAC Treaty signed in Arusha	Arusha, Tanzania (https://www.eac.int)	21st Ordinary Summit in 2021 (online)	EASTECO's Strategic Plan (2017/18–2022) (in process of adopting an East African STI Policy)	East African Science and Technology Commission (EASTECO) established in 2007, but the operationalization of the Commission was finalized only in 2015 http://easteco.or
Economic Community of Central African States ECCAS (10)	1983 Treaty signed in Libreville (revitalized in 1998)	Libreville, Gabon (https://ceeac-eccas.org)	18th Ordinary Summit in 2020	No	Sub-Department (Direction Education, Culture and Technological Development)
Economic Community of West African States ECOWAS (15)	1975 ECOWAS Treaty in Lagos (expansion of scope and powers in 1993)	Abuja, Nigeria (https://www.ecowas.int)	58th Ordinary Summit in 2021 (online)	ECOWAS Policy on Science and Technology (ECOPOST) in 2011	Directorate of Education, Culture, Science and Technology, https://www.esc.comm.ecowas.int/all-about-esc/
Intergovernmental Authority on Development IGAD (8)	1996 Agreement, launched in Djibouti	Djibouti, Djibouti (https://igad.int)	36th Extraordinary Assembly in 2020 (online)	Regional Education Policy Framework, with priority/area 5.4: STI and Indigenous Knowledge (2020)	Governance Structure for Regional Education and STI (ESTI) Programme to be established
Southern African Development Community SADC (15)	1992 Treaty of SADC signed in Windhoek (amended in 2001)	Gaborone, Botswana (https://www.sadc.int)	40th SADC Ordinary Summit in 2020 (online)	SADC Protocol on STI in 2008	Science Technology and Innovation Desk, launched in 2008

Sources: Authors' elaboration from the African Union's website (RECs section), AU (2019), and official RECs' webpages (see Appendix B).

Notes: EAC: initially signed by Kenya, Uganda, and Tanzania; then by Rwanda and Burundi in 2007; AMU is not a signatory to the protocol between the RECs and the AU.

The next section covers the determinants of scientific collaboration as underlined by previous bibliometric studies. Also, it looks further into the patterns of the existing regional and international co-publications of REC member states in the most recent period.

### 2.2 International co-publications in Africa: determinants and regional performances (2014–19)

There exists a dedicated stream of literature that relies upon co-publications as a proxy for research collaborations. It mostly exploits the proximity approach in order to identify patterns in the scientific and technological collaborations across cities, countries, and regions. The academic studies cover groups of advanced economies (Cassi et al., 2015; Hardeman et al., 2015; Hoekman et al., 2010; Ponds et al., 2007; Picci, 2010) and emerging economies (Montobbio and Sterzi, 2013; Scherngell and Hu, 2011; Finardi and Buratti, 2016; Hu et al., 2020).

Over the last two decades, the use of bibliometric data has been expanding in the literature dedicated to the measurement of African research output and its impact (see for instance the works of Arvanitis et al., 2000; Tijssen, 2007; Pouris and Pouris, 2009; Boshoff, 2009, Boshoff, 2010; Irikefe et al., 2011; Toivanen and Ponomariov, 2011; Mêgnigbêto, 2013; Pouris and Ho, 2014; Confraria and Godinho, 2015; and Sooryamoorthy, 2018). The current patterns and outputs of scientific collaborations in Africa clearly suggest that African countries are still far from fully exploiting the great potential from the synergies and complementarities that exist across national research systems. Nevertheless, research collaborations are on the rise and some signs of (local) growing self-reliance and changing local dynamics are visible (Adams et al., 2014; Guns and Wang, 2017). Moreover, >30 African countries have extended their intra-continental cooperation in the past decade (African Observatory in Science Technology and Innovation, AOSTI, 2014).

The prior analyses focusing on samples of African countries have suggested that the intensity of scientific collaboration varies, depending on a number of factors, including the existence of a common language, shared history, and geographical “proximity” (see Mêgnigbêto, 2013, for an overview of West African collaborations; also, Adams et al., 2014). As underlined by Adams and co-authors, “collaboration is driven partly by geography but also by shared culture and – very strongly – by language” (Adams et al., 2014, p. 554). However, as suggested by the authors (ibid.), the lack of dedicated research or regular monitoring of scientific activities in Africa prevents a better understanding of the context-specific nature of the collaborations that do occur and of their changing bottom-up and local drivers. Such evidence is instrumental to inform existing and flourishing policies and plans aimed at regional scientific integration and at building synergies across the continent.

In the African context, the influence of international partnerships continues to constitute a key driving force in terms of the funding, agenda, and priority setting. Also, while international collaboration with non-African countries can be seen as an indicator of scientific quality, a high share of ‘outside’ collaboration “may denote a situation of dependence” on external resources (AOSTI, 2014, p. xvi). Other factors at play relate to Francophone–Anglophone differences and the related cultural and historical legacies. These differences can matter greatly in determining the evolution of scientific collaborations on the continent (Adams et al., 2010, and Boshoff, 2009, Boshoff, 2010, on central and southern Africa; Mêgnigbêto, 2013, for western Africa). Ultimately, the emergence of integrated regional systems will likely entail in the long run a decrease in the importance of external partners in shaping African collaborations, since local capabilities would then be constructed and should rise to the fore.

The present paper builds upon these previous findings. It examines the regional factors that have contributed to shaping scientific collaboration in Africa, together with the influence of historical ties over time. A key rationale is that regional-specific effects should be increasing as the integration efforts are intensifying. However, the RECs show a varying picture of joint efforts and advancements in the area of STI policy, as illustrated in the previous section. Under our framework, these differences should translate into different magnitudes and levels of significance of the RECs or in their relative importance in shaping the patterns of African regional scientific collaboration.

The following graphs illustrate the patterns of scientific collaborations for each REC. These were elaborated from the Web of Science's publications dataset and cover the international co-publications recorded for each country member in the RECs.

**Fig. 1:**
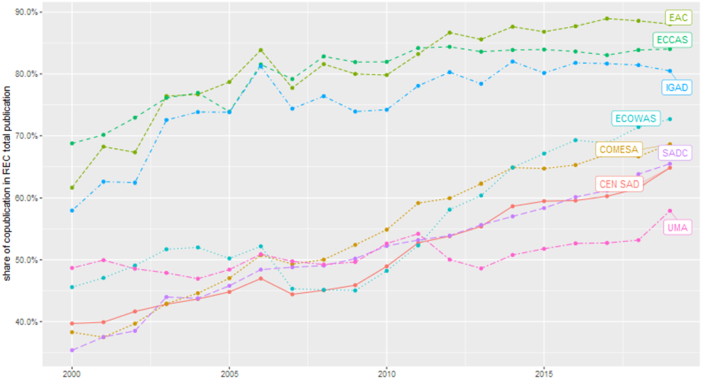
Evolution of the share of co-publications in the total publications by Regional Economic Community (REC). Source: Authors' elaboration from Thomson Reuters' Web of Science (WoS) database - science citation index expanded.

Fig. 1 shows the time trends for the share of co-publications over the total publications from the selected REC's country members.

**Fig. 2:**
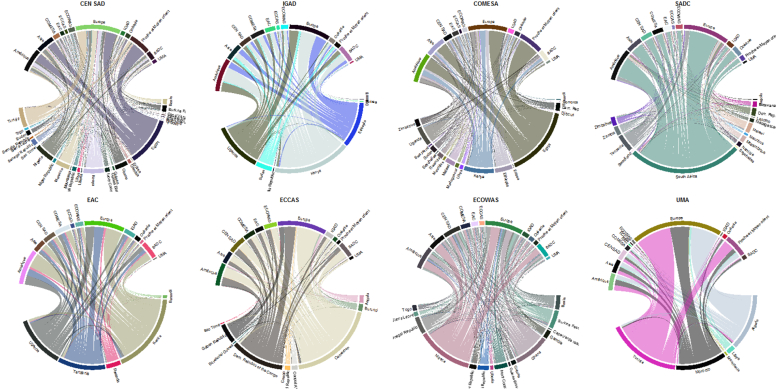
International co-publications of the RECs in Africa (2014–19). Source: Authors' elaborations from Thomson Reuters' WoS database - Sci expanded. Note. The graphs were elaborated with R-4.0.1. The bottom part of the circle includes only the countries that belong to the selected REC.

Fig. 2 shows the patterns for the regional and international publications of the RECs. The top half shows the countries that are not members of the REC, while the bottom half includes all the member states of the selected REC. The arcs link these two groups according to the intensity of co-publications across countries and regional groupings.

Fig. 1 confirms the increasing trend in the number of scientific collaborations as captured by the publication data over the last 20 years. In the most recent years, about 55 % or more of the RECs' publications involve collaborations, either with international partners or with other African countries. This share is even above 80 % for the East African Community (EAC), the Economic Community of Central African States (ECCAS), and the Intergovernmental Authority on Development (IGAD).

Fig. 2 shows the patterns for the RECs' international co-publications. It confirms that all the RECs' member states undertake international collaboration within and outside their specific REC and even beyond the continent. Nevertheless, outward-looking collaborations clearly prevail in all the RECs. A few major intra-REC poles emerge that are worth highlighting, including:

- Egypt for CEN-SAD and COMESA (the two most numerous communities),
- Kenya, Ethiopia, and Uganda for IGAD,
- South Africa for SADC,
- Cameroon and Democratic Republic of Congo for ECCAS, and
- Nigeria and Ghana for ECOWAS.

Most of these countries have indeed already been identified as major publishers on the continent (see Tijssen, 2007; Pouris and Ho, 2014). Tunisia, Algeria, and Morocco are the main actors in scientific collaboration within the UMA. In this regional community, Europe is the main partner in >50 % of the international co-publications. Note, however, that a few bilateral relations, such as those involving Egypt, Algeria, and Tunisia, can be observed with the Near and Middle East, although Europe and the Americas remain the top continental partners; a similar pattern can be seen for other co-publications involving Asian countries with Egypt, Kenya, South Africa, and Nigeria. Furthermore, even if all countries are involved in some collaboration, the density of within-community collaborations significantly differs across the RECs.

## 3 Methodology and data

### 3.1 The proximity approach: main rationale and relevance

In order to understand the determinants of scientific collaboration across countries in Africa, we draw from a stream of studies in spatial scientometrics (Frenken et al., 2009). This stream builds upon the proximity approach developed in regional studies (Boschma, 2005) and offers a comprehensive framework to analyse the scientific collaboration among economic actors. A key finding is that personal and organisational interactions are influenced by different dimensions of proximity, in particular the interactions that involve the exchange of intangible goods, such as scientific knowledge. Proximity between actors may refer here to its geographical, cognitive (or scientific), social, organisational, or institutional dimensions. One rationale for the proximity approach resides, for instance, in the fact that a greater proximity between researchers should facilitate the sharing of tacit knowledge (Frenken et al., 2009). This in turn can contribute to a broader integration of scientific communities, since researchers would tend to read each other's scientific works, exchange information, and collaborate, which would promote research mobility and research stays.

Our empirical application considers four dimensions, including the cognitive (or scientific), geographical, social, and institutional proximity dimensions, to explain the co-publication activities between two countries.^5^ Cognitive proximity refers to the differences in the mind-set and knowledge domain of actors. On the one hand, a certain degree of cognitive similarity is often needed to understand each other and to benefit from interactions; on the other hand, some cognitive dissimilarity is relevant in the search for complementary knowledge. Following Cassi et al. (2015), we capture the cognitive proximity by calculating the cosine similarities between the scientific profiles of two given countries. Besides the cognitive proximity, scientists can also share some social traits, thus facilitating the establishment of trust and reducing the likelihood of opportunistic behaviours among the collaborating actors. Our study considers a direct and an indirect proxy here. The direct proxy is the number of past collaborations between two countries; while the indirect one is the number of direct partners that the two countries have in common. This latter proxy is related to the fact that scientific collaboration with a co-author of existing co-authors is more likely.

Finally, we analyse how institutional proximity, or similarity in the “rules of the game”, influences African scientific collaboration. Since partners are aware of the prevailing formal and informal rules, aligning their incentive systems should be relatively easier. On the contrary, dissimilar institutional contexts can generate misunderstanding and misalignment of the incentive systems, which in turn would discourage collaboration. In our framework, this dimension of proximity between two countries is captured here through the existence of a common colonial heritage and the use of a common language. Adding to the literature, we also consider the common institutional membership or whether two African countries belong to the same regional economic community (REC) or to the African Malagasy Higher Education Council (CAMES).

### 3.2 Econometric approach

In line with previous empirical studies on scientific and technological collaborations, we modelled the co-publication patterns between any two countries using a gravity model (Ponds et al., 2007; Hoekman et al., 2010; Montobbio and Sterzi, 2013). Initially exploited to explain inter-country trade flows (Bergstrand, 1985), the gravity approach takes inspiration from Newton's law of universal gravitation. According to the Newtonian law, the attraction between two objects depends positively on their masses and negatively on their distance. In our conceptualisation, this implies that the intensity of co-publication activity between two countries would increase with their individual number of publications (their mass), and decrease with their distance (for example, geographical distance apart).

Our gravity model can be represented by the following equation:

(1) $${\text{Copub}}_{\mathrm{ijt}}=\exp\;\left[\beta_{0}+\beta_{1}A_{\mathrm{it}}+\beta_{2}A_{\mathrm{jt}}+\beta_{3}{\text{PROX}}_{\mathrm{ijt}}+\beta_{4}{\mathrm{REC}}_{\mathrm{ijt}}+\pi_{i}+\rho_{j}+\lambda_{t}\right]\times \varepsilon_{\mathrm{ijt}}$$

where *Copub*_*ijt*_ is the number of co-publications between countries *i* and *j*, at time *t*. Differently from trade data, the dependent variable is an un-directed one, i.e. *Copub*_*ijt*_ = *Copub*_*jit*_ for any *i* and *j*.^6^

*A*_*it*_ and *A*_*jt*_, respectively, represent some time-variant dimension (i.e. the masses in the gravity equation, such as for instance the number of publications) of countries *i* and *j* at time *t*.

Differently from a standard gravity model and according to the adopted analytical framework, we define relational variables in terms of proximities rather than distances, i.e. PROX_ijt_. For instance, we take the inverse of the geographical distance to obtain the geographical proximity. The implication of this declination is interpretative rather than statistical, that is, in terms of the significance of the results.

REC is a dummy variable informing if the two countries are members of at least one REC in the period or year *t*. *π*_*i*_, *ρ*_*j*_, *λ*_*t*_ are fixed terms for country *i*, country *j*, and year, respectively. *ε*_*ijt*_ measures the error term.

The gravity model in Eq. (1) can be estimated using different econometric techniques. In a seminal article, Santos Silva and Tenreyro (2006) show that a log linear model provides biased estimates of mean effects when the errors are heteroscedastic. In order to address this issue, the authors suggest the use of the Poisson pseudo-maximum likelihood (PPML) estimator as a robust alternative. Moreover, this estimator solves the problem of a zero value of the dependent variable and fits well for count data, as in our case. Indeed, it allows dealing with zero overdispersion as shown in a most recent article (Santos Silva and Tenreyro, 2011). Our dataset displays a rate of 60 % zero values in the period 2002–2019. This average value decreases over time, from 81 % in 2002 down to 30 % in 2019.

We performed two sets of regressions: the first one included all the years from 2002 to 2019, and the second set broke down the sample into three sub-periods to grasp changes in the determinants. In all the regressions, we controlled for the fixed effects at both the country and time levels (Baldwin and Taglioni, 2006). Moreover, robust standard errors were clustered to control for error correlation in the panel, since the observations in pairs of countries are likely to be dependent across years.

### 3.3 Data and variables

The main source of data for our study is the HCERES-OST scientific publications dataset, an enriched^7^ version of Thomson Reuters' Web of Science (WoS) database - science citation index expanded (henceforth WoS).

Despite the use of an enriched version of the WoS, the data suffers from the same drawbacks as the original data in terms of coverage.^8^ First, in terms of scientific fields: the WoS has a bias towards hard sciences, while the social sciences and humanities are highly underrepresented. Second, de facto mainly scientific publications in English are reported and many national journals are not considered, and this is especially true for developing countries (Rafols et al., 2016). However, the WoS and Scopus information about international collaboration seems to correlate well with the actual international scientific publications of African researchers, according to Boshoff and Akanmu (2017). Nevertheless, the latter authors' domain-specific analysis of a Nigerian university revealed the existence of a bias related to international collaborations that involve exclusively African countries. These latter ones are underrepresented when compared with those collaborations including at least one non-African partner. On the one hand, this bias implies that international comparisons between African and non-African scientific activities can be misleading; but, on the other hand, it also implies that comparing African countries among themselves using this kind of source should be less problematic (Boshoff and Akanmu, 2017). Finally, worldwide datasets, such as the WoS, have at least the advantage of quality filters, whereas other datasets cannot guarantee the same (Google Scholar for instance).

The sample covers a subset of works published in 11 scientific fields^9^ between 2002 and 2019, reporting among other items the authors' affiliations, which were located in 53 African countries. This means that the initial sample concerned all publications involving at least one author affiliated in one of the 53 African countries and that we have information on the collaborations with authors located outside of the continent. Note, Gambia and South Sudan were excluded, essentially because the country code of Gambia was not available at the time of the download while only five years of date were available for South Sudan (the country had only obtained its independence (from the North) in 2011). A complementary source of data is the Cepii Gravity Dataset (Head and Mayer, 2014).

Table 2 reports the definitions of the variables for the gravity equation, while Table 3 reports the basic descriptive statistics.^10^

**Table 2:** List of variables and their definitions.

Variable	Definition
copub_ijt_	The number of scientific paper co-authored by residents of countries i and j at time t
lang_eth_ij_	*i* and *j* share a common language (dummy)
comcol_ij_	*i* and *j* have ever had a colonial heritage (dummy)
geo_prox_ij_	The inverse of *i* and *j* geographical distance weighted by population of main agglomeration (logarithm)
logpastcopub_ijt_	Stock of co-publication between country *i* and *j*, time *t-1* to *t-3* (logarithm)
logpub_it_	Stock of publication of country *i*, time *t-* to *t-3*(logarithm)
rec_ijt_	*i* and *j* belong to a same REC at time t (dummy)
CAMESij	i and j belong to CAMES
CEN_SAD_ijt_ (etc.)	i and j belong to a specific REC at time t (8 dummies)
science_prox_ijt_	Cosine similarity of the scientific profiles of country's *i* and *j*, time *t-1* to *t-3*. The scientific profile is proxy by the distribution of the shares of publications by field.
logEU_ijt_	Number of EU countries scientific partners between countries *i* and *j*, time *t-1* and *t-3* (logarithm)

**Table 3:** Descriptive statistics.

Variable	Obs	Mean	Std. dev.	Min	Max
geo_prox	1378	−8.029417	0.6560399	−9.187282	−5.088719
comcol	1378	0.2583454	0.4378838	0	1
lang_eth	1378	0.3222061	0.4674911	0	1
cames	1378	0.1240929	0.3298072	0	1
copub	24,804	3.996009	17.03928	0	870
logpub_i	24,804	5.487753	2.46161	0	10.92756
logpub_j	24,804	5.101726	2.40463	0	10.92756
logpastcopub	24,804	0.8075626	1.21263	0	7.167809
logeu	24,804	1.059752	1.326256	0	3.332205
science_prox	24,804	0.7811336	0.2104682	0	0.9996395
dummy_cer	24,804	0.4198113	0.4935378	0	1
cen_sad	24,804	0.2075875	0.4055879	0	1
comesa	24,804	0.1092163	0.3119166	0	1
eac	24,804	0.0058458	0.0762358	0	1
eccas	24,804	0.0346718	0.1829509	0	1
ecowas	24,804	0.0761974	0.265319	0	1
igad	24,804	0.0120948	0.1093116	0	1
sadc	24,804	0.0655136	0.247435	0	1
uma	24,804	0.0072569	0.0848795	0	1

For each pair of countries, *i* and *j*, we have some relational variables, which do not vary over time. These include the geographical proximity, the existence of a common colonial heritage, a common language, and a common CAMES membership. For these variables, we have only n × (n-1)/2 unique observations (1378). The geographical distance is transformed in terms of proximity considering its inverse (i.e. geo_prox_ij_).^11^ Moreover, in order to proxy the institutional proximity, we set a dummy variable that captures whether two countries share common past colonial ties, and another dummy variable which captures whether the two countries have the same language. For this latter variable, we opt for the second-most important language of the country (that is the ethnic language dummy in the Cepii dataset), because the official one may be redundant or may reflect some information already captured by the common colonial heritage dummy.^12^ Additionally, a dummy variable is included to signal if the two countries belong to the CAMES.

For each dyad, we calculated the variables that can vary over time; for each of these, we had 24,804 observations. The co-publication was the count of the publications co-authored by two countries at time *t* (i.e. copub_ijt_) and past co-publications was the amount of publications co-authored by the two countries in the previous three years (i.e. pastcopub_ijt_ and its logarithm: log pastcopub_ijt_). This variable could proxy the *social* proximity of the two countries, since it measures the existing collaborations. For past co-publications, we calculated the share of publications involving a non-African country. In doing so, we also looked, for each country pair, at the effects of ‘having the same European Union (EU) partner country’.

For each African country *i* at year *t*, we calculated the stock of publications in the previous three years (i.e. publication_it_). Based on this stock, we calculated the scientific profile of the country using their publications in the 11 fields (i.e. vector of shares by field). The procedure was repeated for each country. Then, the scientific proximity between two countries *i* and *j* (science_prox_ijt_) was calculated as the cosine similarity between the scientific profiles (or vectors) of the two countries.

Finally, we had eight dummies reporting whether the two countries belong to a specific Regional Economic Community. Membership of a REC was not exclusive since a given country can hold several community memberships; however, each country participated in at least one REC. Although it is relatively uncommon, some shifts in REC membership can occur over time. A summarising dummy reported the information if two countries had at least one common REC; this was the case for one dyad out of two, as reported in Table 3.

### 3.4 Co-publication patterns of Regional Economic Communities, 2002–2019

The 53 African countries in the sample had published about 668,597 distinct publications, which included 368,516 international co-publications between 2002 and 2019. Out of these 368,516 international co-publications 30,824 (or 8.4 %) involved at least two African countries. In the period of observation, the continent share increased to about 3.25 % of the world's total scientific publications and 2.83 % of the co-publications in 2019 (see also Table 4).

**Table 4:** Scientific publications and co-publications of Africa-based researchers.

Year	Nb of publications	Nb of co-publications	Nb of co-publications with at least 2 Africa-based researchers	World's total publications	World's total co-publications	% of world's publications	% of world's co-publications	Share of co-publications w. at least 2 African countries over African total co-publications
2002	14,442	5890	344	969,812	445,278	1.5 %	1.3 %	6 %
2003	15,019	6618	389	1,031,442	484,540	1.5 %	1.4 %	6 %
2004	15,981	7134	410	1,081,680	521,887	1.5 %	1.4 %	6 %
2005	16,787	7716	487	1,145,755	557,927	1.5 %	1.4 %	6 %
2006	19,535	9547	634	1,216,404	667,917	1.6 %	1.4 %	7 %
2007	23,249	10,970	770	1,328,028	734,662	1.8 %	1.5 %	7 %
2008	26,158	12,404	895	1,419,613	793,640	1.8 %	1.6 %	7 %
2009	29,661	14,368	1113	1,496,660	851,027	2.0 %	1.7 %	8 %
2010	31,449	15,973	1252	1,525,952	893,431	2.1 %	1.8 %	8 %
2011	34,726	18,486	1574	1,610,067	968,969	2.2 %	1.9 %	9 %
2012	37,954	20,230	1630	1,716,798	1,035,796	2.2 %	2.0 %	8 %
2013	42,778	23,456	1823	1,800,473	1,120,811	2.4 %	2.1 %	8 %
2014	47,981	27,402	2119	1,887,863	1,212,915	2.5 %	2.3 %	8 %
2015	53,347	30,810	2531	1,961,589	1,296,117	2.7 %	2.4 %	8 %
2016	59,321	34,868	3061	2,046,158	1,381,099	2.9 %	2.5 %	9 %
2017	63,853	37,985	3409	2,104,282	1,442,882	3.0 %	2.6 %	9 %
2018	66,960	40,644	3876	2,132,645	1,502,928	3.1 %	2.7 %	10 %
2019	69,396	44,015	4507	2,134,200	1,556,327	3.3 %	2.8 %	10 %
Total publications 2002–2019	668,597	368,516	30,824	28,609,421	17,468,153	2.3 %	2.1 %	8 %

The three first columns of Table 4 provide, for each year, the number of African publications and co-publications, and the number of African co-publications involving at least two African countries, respectively. The three final columns show the corresponding shares in the world's total publications and co-publications, and the share of the publications involving at least two African countries in Africa's total co-publications.

Besides the overall upward trends, continuing important efforts are still focused on increasing the continental collaboration. Indeed, the number of collaborations involving at least two African countries increased by >13 times between 2002 and 2019. This is twice more than the increase observed in the number of co-publications involving at least one African country. These trends are also visible in the number of countries participating in scientific collaboration: only 19 % of possible bilateral collaborations could be observed at the beginning of the period, while 70 % of possible collaborations had occurred by the end of the period.

The great variability observed within the RECs reflects the heterogeneous characteristics and endowments of the national science and research systems of the member countries. In spite of the increasing publication activity, Africa still suffers from low domestic capacities in terms of human resources, STI funding, and economic policies as well as from critical institutional and structural gaps (see Irikefe et al., 2011, and AOSTI, 2013, for country-specific gaps, and Allard and Williams, 2020, for a discussion on the drivers of national-level innovation in 39 African countries).

Table 5 gives a few statistics for individual RECs for three selected periods of six years between 2002 and 2019. The left-hand columns show the number of publications, the percentage of domestic publications, and the percentage of publications in the top 10 % of the most-cited publications. The improvement in highly performing research in CEN-SAD, COMESA, ECCAS, ECOWAS, SADC, and UMA came with a decrease in exclusively domestic co-publications. These overall improvements in the quality research were also pointed out by Blom et al. (2016) and Dosso et al. (2017), among others.

**Table 5:** Main features of the publications by the RECs.

REC	PERIODE	Publications			Co-publications			
		NB_PUB	% of domestic publications	Share of publications in Top 10 %	% of co-publications with African countries outside the REC (in total co-publications)	% of co-publications with non-African countries (in total co-publications)	% of co-publications excl. between the countries of the REC (in total co-publications)	% of international co-publications involving at least another country of the REC
CEN SAD (29)	2002–07	54,813	55.8 %	6.3 %	2.22 %	96.67 %	1.11 %	4.35 %
CEN SAD	2008–13	86,116	50.3 %	6.8 %	2.92 %	95.84 %	1.23 %	5.18 %
CEN SAD	2014–19	200,914	39.0 %	7.9 %	3.47 %	95.65 %	0.88 %	5.29 %
COMESA (18)	2002–07	33,619	53.9 %	7.5 %	3.54 %	95.71 %	0.75 %	3.57 %
COMESA	2008–13	52,256	44.1 %	8.6 %	3.48 %	95.77 %	0.75 %	4.32 %
COMESA	2014–19	131,782	33.6 %	9.2 %	3.20 %	96.26 %	0.54 %	4.60 %
EAC (5)	2002–07	7886	22.7 %	11.4 %	4.35 %	94.47 %	1.18 %	5.77 %
EAC	2008–13	12,047	17.5 %	12.6 %	4.93 %	94.11 %	0.96 %	7.03 %
EAC	2014–19	27,449	12.0 %	11.0 %	5.99 %	93.17 %	0.84 %	8.53 %
ECCAS (10)	2002–07	3276	22.8 %	6.5 %	6.05 %	93.52 %	0.43 %	3.64 %
ECCAS	2008–13	4684	16.9 %	7.2 %	8.68 %	90.73 %	0.59 %	4.88 %
ECCAS	2014–19	10,675	16.3 %	8.8 %	8.35 %	90.99 %	0.66 %	6.27 %
ECOWAS (15)	2002–07	12,986	50.3 %	5.5 %	5.27 %	92.64 %	2.09 %	8.72 %
ECOWAS	2008–13	20,950	50.0 %	5.5 %	8.15 %	89.89 %	1.96 %	9.72 %
ECOWAS	2014–19	43,548	30.5 %	7.7 %	9.56 %	89.03 %	1.41 %	9.72 %
IGAD (7)	2002–07	8474	26.5 %	10.4 %	5.23 %	93.87 %	0.90 %	4.22 %
IGAD	2008–13	13,279	23.2 %	11.1 %	5.38 %	93.98 %	0.64 %	5.44 %
IGAD	2014–19	33,569	18.8 %	9.8 %	5.97 %	93.47 %	0.56 %	6.36 %
SADC (15)	2002–07	40,124	54.7 %	8.7 %	2.85 %	95.21 %	1.94 %	4.96 %
SADC	2008–13	57,307	48.0 %	9.6 %	4.21 %	93.42 %	2.37 %	6.62 %
SADC	2014–19	122,461	38.7 %	9.9 %	5.55 %	92.06 %	2.38 %	8.12 %
UMA (5)	2002–07	21,998	51.1 %	5.4 %	0.71 %	98.74 %	0.56 %	1.91 %
UMA	2008–13	36,969	48.8 %	5.8 %	1.33 %	97.84 %	0.84 %	2.65 %
UMA	2014–19	87,052	46.7 %	5.9 %	1.34 %	97.52 %	1.14 %	3.48 %

Source: Authors' elaborations from Thomson Reuters' WoS – Sci expanded.

Notes: (1) NB_PUB denotes the number of citable documents. (2) For the top 10 %, the number of publications is normalised by year and discipline. The first three columns of the co-publications sum up to 100 %.

The right-hand columns of Table 5 give additional details on the decomposition of the co-publications by REC. The share of international co-publications with African countries outside the specific REC reflects the extent to which the REC, as an entity, collaborates with other African countries. Except for COMESA, all the regional communities showed an increase for this indicator. However, the overwhelming share of international co-publications with non-African countries showed only slightly decreasing trends; this holds true for six out of the eight RECs. As mentioned in the previous section, increasing international collaboration can be interpreted as an indicator of scientific quality, but a high share of ‘outside’ collaboration may also reflect a situation of dependence (AOSTI, 2014, p. xvi). As for the share of co-publications within REC members, only SADC, and to a lower extent ECCAS, showed an opposite trend. In other words, the proportion of intra-REC collaboration showed an increase in these two communities. Nevertheless, all the RECs showed improvement to a different extent in the share of international co-publications that involved at least one other country from the same REC. Additionally, Table C.1, Table C.2 in Appendix C provide similar statistics by scientific discipline for each REC in the period 2014–2019,^13^ and they confirm the heterogeneity observed across the RECs. They also underline the relevance of accounting for the country scientific profiles or proximity in the identification of the factors driving scientific collaboration.

The observations made in this sub-section suggest that the influence of international actors still prevails in the development of African scientific collaboration. They also suggest that REC-specific effects may be at play together with an emerging continental integration effect (the role of other African countries outside the REC).

This paper investigates these regional factors, along with traditional drivers of scientific collaboration. In doing so, it also provides a first and original assessment of the additional effect of the RECs on scientific integration in Africa, especially considering their legal mandate to foster regional STI policy alignment and cooperation, as a first step towards broader continental integration (see Section 2). The next section presents the results of our econometric investigations.

## 4 Empirical results

Table 6, Table 7 provide the main results of our empirical application. Table 6 gives the estimates for the baseline model and Table 7 reports the econometric results for the effects of ‘belonging to a specific regional community’ over time.

**Table 6:** Estimates of the baseline model, Poisson pseudo-maximum likelihood, with co-publication as the dependent variable.

	(1)	(2)	(3)	(4)	(5)	(6)
logpub_i	−0.0564	−0.0474	−0.0471	−0.0560	−0.0526	−0.0442
	(0.0360)	(0.0361)	(0.0362)	(0.0361)	(0.0359)	(0.0359)
logpub_j	0.00979	0.0183	0.0188	0.0103	0.0131	0.0207
	(0.0383)	(0.0382)	(0.0382)	(0.0382)	(0.0384)	(0.0383)
geo_prox	0.223⁎⁎⁎	0.236⁎⁎⁎	0.250⁎⁎⁎	0.238⁎⁎⁎	0.221⁎⁎⁎	0.236⁎⁎⁎
	(0.0169)	(0.0162)	(0.0232)	(0.0242)	(0.0259)	(0.0250)
logpastcopub	0.518⁎⁎⁎	0.500⁎⁎⁎	0.499⁎⁎⁎	0.517⁎⁎⁎	0.512⁎⁎⁎	0.495⁎⁎⁎
	(0.0188)	(0.0190)	(0.0190)	(0.0189)	(0.0184)	(0.0184)
comcol	0.107⁎⁎⁎	0.0298	0.0317	0.109⁎⁎⁎	0.105⁎⁎⁎	0.0294
	(0.0275)	(0.0282)	(0.0284)	(0.0278)	(0.0271)	(0.0278)
science_prox	1.819⁎⁎⁎	1.878⁎⁎⁎	1.861⁎⁎⁎	1.801⁎⁎⁎	1.952⁎⁎⁎	1.972⁎⁎⁎
	(0.138)	(0.139)	(0.140)	(0.139)	(0.170)	(0.168)
lang_eth	0.242⁎⁎⁎	0.193⁎⁎⁎	0.191⁎⁎⁎	0.240⁎⁎⁎	0.263⁎⁎⁎	0.209⁎⁎⁎
	(0.0361)	(0.0336)	(0.0337)	(0.0360)	(0.0319)	(0.0308)
logeu	0.173⁎⁎⁎	0.174⁎⁎⁎	0.174⁎⁎⁎	0.173⁎⁎⁎	0.172⁎⁎⁎	0.173⁎⁎⁎
	(0.0175)	(0.0174)	(0.0174)	(0.0175)	(0.0173)	(0.0173)
cames		0.390⁎⁎⁎	0.390⁎⁎⁎			0.375⁎⁎⁎
		(0.0603)	(0.0603)			(0.0574)
dummy_cer			−0.0267	−0.0275		
			(0.0261)	(0.0272)		
cen_sad					−0.0169	0.0134
					(0.0525)	(0.0481)
comesa					0.0848⁎	0.0950⁎⁎
					(0.0360)	(0.0363)
eac					−0.0324	0.00325
					(0.0576)	(0.0588)
eccas					0.310⁎⁎⁎	0.253⁎⁎⁎
					(0.0584)	(0.0583)
ecowas					0.0248	−0.0270
					(0.0694)	(0.0612)
igad					0.0523	0.0180
					(0.0451)	(0.0466)
sadc					−0.0643	−0.0690
					(0.0465)	(0.0439)
uma					−0.156	−0.101
					(0.114)	(0.105)
cons	0.670	0.778	0.910⁎	0.806	0.475	0.636
	(0.433)	(0.435)	(0.459)	(0.461)	(0.470)	(0.465)
N	24,786	24,786	24,786	24,786	24,786	24,786

Countries *i* and *j* fixed effects and year fixed effects.

Country-pair clustered robust standard errors are reported in parentheses.

⁎ *p* < 0.05.

⁎⁎ *p* < 0.01.

⁎⁎⁎ *p* < 0.001.

**Table 7:** Estimates of REC effects, Poisson pseudo-maximum likelihood, with co-publication as a dependent variable.

	(1)	(2)	(3)	(4)	(5)	(6)
	2002–07	2008–13	2014–19	2002–07	2008–13	2014–19
logpub_i	−0.0645⁎	−0.204	−0.151	−0.0620⁎	−0.205	−0.147
	(0.0300)	(0.113)	(0.0964)	(0.0293)	(0.113)	(0.0957)
logpub_j	−0.150⁎⁎⁎	−0.0499	0.0490	−0.148⁎⁎⁎	−0.0457	0.0541
	(0.0355)	(0.138)	(0.0951)	(0.0341)	(0.139)	(0.0943)
geo_prox	0.616⁎⁎⁎	0.315⁎⁎⁎	0.172⁎⁎⁎	0.587⁎⁎⁎	0.295⁎⁎⁎	0.165⁎⁎⁎
	(0.0628)	(0.0429)	(0.0202)	(0.0668)	(0.0431)	(0.0220)
logpastcopub	0.221⁎⁎⁎	0.526⁎⁎⁎	0.568⁎⁎⁎	0.205⁎⁎⁎	0.519⁎⁎⁎	0.559⁎⁎⁎
	(0.0412)	(0.0281)	(0.0209)	(0.0384)	(0.0276)	(0.0198)
comcol	0.435⁎⁎⁎	0.112⁎	−0.0308	0.413⁎⁎⁎	0.119⁎⁎	−0.0286
	(0.0762)	(0.0440)	(0.0261)	(0.0814)	(0.0448)	(0.0256)
science_prox	2.391⁎⁎⁎	2.326⁎⁎⁎	1.501⁎⁎⁎	2.109⁎⁎⁎	2.351⁎⁎⁎	1.734⁎⁎⁎
	(0.251)	(0.209)	(0.138)	(0.322)	(0.230)	(0.127)
lang_eth	0.339⁎⁎⁎	0.219⁎⁎	0.141⁎⁎⁎	0.354⁎⁎⁎	0.246⁎⁎⁎	0.159⁎⁎⁎
	(0.0775)	(0.0767)	(0.0268)	(0.0838)	(0.0672)	(0.0261)
cames	0.388⁎⁎	0.271⁎	0.387⁎⁎⁎	0.338⁎	0.225	0.375⁎⁎⁎
	(0.151)	(0.132)	(0.0509)	(0.157)	(0.116)	(0.0512)
dummy_cer	−0.00601	−0.0460	−0.0251			
	(0.0639)	(0.0497)	(0.0246)			
logeu	0.0809⁎⁎	0.232⁎⁎⁎	0.0644⁎⁎	0.0821⁎⁎	0.234⁎⁎⁎	0.0628⁎⁎
	(0.0295)	(0.0310)	(0.0205)	(0.0295)	(0.0316)	(0.0203)
cen_sad			−0.0758	−0.0407	0.0394	
				(0.0813)	(0.126)	(0.0423)
comesa				0.0505	0.0500	0.0870⁎⁎
				(0.104)	(0.0713)	(0.0312)
eac				0.0153	−0.122	0.00316
				(0.166)	(0.108)	(0.0485)
eccas			0.349⁎	0.313⁎⁎	0.216⁎⁎⁎	
				(0.155)	(0.0978)	(0.0606)
ecowas			0.0295	0.118	−0.0558	
				(0.152)	(0.138)	(0.0530)
igad				0.128	0.0429	0.00268
				(0.142)	(0.0798)	(0.0394)
sadc			0.0427	−0.134	−0.0613	
				(0.119)	(0.0788)	(0.0390)
uma			0.757⁎	0.0269	−0.198⁎	
				(0.324)	(0.208)	(0.0857)
_cons	3.758⁎⁎⁎	1.653	1.338	3.753⁎⁎⁎	1.500	0.980
	(0.595)	(1.341)	(1.117)	(0.685)	(1.292)	(1.110)
N	7782	8004	8262	7782	8004	8262

Countries *i* and *j* fixed effects and year fixed effect.

Country-pair clustered robust standard errors are reported in parentheses.

⁎ p < 0.05.

⁎⁎ p < 0.01.

⁎⁎⁎ p < 0.00.

The first specification reported in Table 6, i.e. specification (1), corresponds to the basic gravity specification, including the attributes of the countries and some relational variables. The country attributes were not statistically significant due to the country fixed effects. Conversely, all the relational variables had a significant and positive sign. This could be expected since they were defined by proximity.

Generally, the more similar two countries are in terms of their scientific profiles, the higher the intensity of co-publications that can be expected. The impact of this proximity dimension seems to be quite important for African collaboration. Collaboration between very different disciplines usually requires geographical and institutional proximity in order to overcome difficulties related to the existence of a low cognitive proximity. Considering the complementarities among the proximity dimensions, a high coefficient of scientific proximity can also signal that these complementarities might not be at play in the African collaboration context.

‘Speaking the same language’ also matters, as suggested by the descriptive and mapping analyses of Mêgnigbêto (2013) and Adams et al. (2014). The positive effect of past co-publications means that two countries are more likely to collaborate if they have previously collaborated. Indeed, future collaborations can benefit from existing communication channels (and even established routines) and frameworks (institutional, scientific, etc.). At the policy level, this perspective can guide the setting up and delivery of additional support, especially for emerging networks and for the initiation of collaborations with other non-partner African countries with similar research interests (where scientific proximity matters) to enable the development of sustainable collaboration networks.

Moreover, the importance of third partner countries in African scientific collaboration was captured by the number of EU countries that two African countries have as common partners (or co-authors) in a given time period. In this regard, France ranked second on the list of top non-African partners^14^ in African co-publications while the UK^15^ ranked third, suggesting a still significant role of historical heritage. This is also consistent with the significant effect related to the dummy variable for reporting a common colonial link after the year 1945.

Specifications (2) to (4) gradually introduce two institutional binary variables that capture the pairs of countries with CAMES membership and with a REC membership. While the CAMES estimate was significant and positive, REC membership did not seem to have an additional significant effect on the likelihood of African countries collaborating. Alternatively, this latter result may reflect the high diversity across and within the regional communities in terms of scientific capability and maturity as well as the limited structuring role the RECs play in African science integration.

The positive and significant coefficient for the CAMES may relate to its historical influence in coordination and scientific cooperation among African Francophone higher education and research systems. The CAMES was initially composed of only French-speaking countries, but was later joined by Guinea-Bissau (2005), Equatorial Guinea (2010), and the Democratic Republic of Congo (2011). With more than a half century of existence, the African and Malagasy Council for Higher Education has certainly reached a certain structuring maturity. Owing to its initial mission of systematic coordination and its role in promoting a culture of scientific cooperation, especially in the harmonisation of curricula, career development, and academic tenure, the CAMES seems to have contributed to the development of a sustained local scientific networking among French-speaking countries. Noteworthily, the recent reforms adopted in Antananarivo in the 2000s have reinforced the levers and the structuring missions of the council. Above all, this result calls for caution and for additional research to better understand the funding and organisational model, challenges, and bottlenecks underlying the 50 years of existence of the CAMES.^16^ Indeed, although the council is legitimated by high-level policy commitment (Ministerial Council), its organisational functioning relies upon senior research and academic experts. Nevertheless, the council suffers from multilevel resource gaps, modernisation challenges, and a surprisingly low visibility considering its potential long-term impact on scientific integration (see Cissé, 2018).

Finally, specifications (5) and (6) estimated whether any of the eight RECs was positively correlated with the number of co-publications between pairs of countries once the gravity determinants, and country and year specificities were controlled for. Specification (6) additionally included a CAMES dummy. As for the individual RECs, the effect was positive and significant only for ECCAS and COMESA.

Table 7 presents six models. They report the regressions for three different sub-periods of models (3) and (6) of Table 6. In doing so, it is possible to verify if the previous results hold true independently of the period considered or if they vary over time.

First, the decreasing magnitude of some coefficients, such as geographical proximity, colonial heritage, and common language, is worth noting. As suggested by Frenken and co-authors, geographical distance apart should become relatively less important in an integrated area (Hoekman et al., 2010), and then other determinants should prevail. Therefore, the decreasing importance of the geographical proximity effect in the African collaboration context could illustrate an increase in integration or at least an intensification of the collaboration network. The same is true for colonial ties and a common ethnical language.

Second, the role of partnership with the European Union seems to have increased at the end of the 2000s. One likely reason for this could be the political impulse given to African–European collaboration under the Joint Africa–EU Strategy (JAES). On the continent, these years also coincided with multiple high-level policy commitments, such as the adoption of Africa's Science and Technology Consolidated Plan of Action 2005–2014 (CPA) and the 2007 Addis Ababa Declaration on Science Technology and Scientific Research for Development, promoting the development of south–south and north–south cooperation.^17^

Concerning the REC dummy variable, we can observe that the ECCAS estimate had a significant positive effect on the three selected periods. Among the likely explanations for this is the overlap with other institutions, such as CAMES; indeed, if we consider all the possible country pairs among the ECCAS members, >62 % are also characterised by common partnership in CAMES. This holds true for only 12 % of the possible dyads across the continent and 34 % for ECOWAS, which reported the second highest value among the RECs (see Table D.1 in the Appendix). COMESA estimates were significant and positive only in the last period, while the signs for UMA's coefficients alternated in between the first and the third periods. Even if these variations raise some questions, the negative significant coefficient may not be a surprising result given the tense relationships among the members of the Arab Maghreb Union (see Parshotam's policy brief for a critical discussion about the AMU integration process, Parshotam, 2020).

## 5 Conclusion

‘African’ collaborative science is on the rise but there is still a long way to go and the path towards an integrated research area in Africa still has many hurdles to overcome. However, the continent is showing positive signals of vibrant local dynamics and emerging networks. The changing research landscape has seen some improvements already, albeit still insufficient, in scientific production and collaboration as well as in the ability of African scientists and organisations to produce high-quality research papers.

Our analysis took inspiration from the proximity analytical framework, which allows distinguishing between different dimensions of proximities in order to explain scientific collaboration between actors. Within this approach, this paper investigated the role of regional factors and regional economic communities together with the influence of historical ties in shaping African scientific collaborations over time.

From a policy perspective, a process of integration will ultimately bring about a process of convergence among countries and an increasing cohesion of the overall structure. In order to evaluate this process, we analysed the main determinants of intra-Africa collaboration. In doing so, we contribute to previous works that have attempted to explain the roles of institutions in facilitating regional collaboration towards scientific integration in Africa. Hence, we provide a first assessment, albeit indirect, of the process of integration of African regional science by investigating the determinants of dyadic collaboration at a country level and the potential integrative role played by the African Regional Economic Communities (RECs) in the area of science collaborations.

Our findings confirm the improvement in regional cohesion, but also highlight a number of acute bottlenecks and challenges, including the need to redefine or rethink some of the policy implementation strategies. Increasing integration is reflected by the decreasing roles of geography proximity, historical heritage, and linguistic ties. Non-African countries, such as European ones, continue to play an important role in supporting collaborations between African countries. These extra regional scientific collaborations seem to play a dual role, since they connect local researchers to the global knowledge pool and also contribute to increasing regional cohesion. Yet, our study could not confirm whether the international influence is too high or say if it denotes a clear situation of dependence (AOSTI, 2014). Nonetheless, our findings echo the recommendations to foster greater domestic capabilities in science in order for African researchers to fully benefit from collaborations with partners located outside of the continent.

The role played by the African RECs is less clear. Together with member states, RECs hold a central role in the local implementation of a continental STI strategy. However in practice, such responsibilities have not yet come with the corresponding resource commitment that are necessary for capacity development. According to our results, only a few of the RECs, such as ECCAS and COMESA, seem to have played an additional role in establishing and supporting collaboration to date, especially in recent years. Furthermore, our results suggest the existence of a long-term structuring role for the African and Malagasy Council for Higher Education (CAMES). While this result deserves additional research, CAMES may offer valuable organisational insights or lessons on how to foster regional scientific integration beyond just the French-speaking countries in Africa. Overall, it underlines that upstream harmonisation and structuring, for instance at the level of the university research curricula, career advancement schemes, and academic tenure, can positively influence sound competition for high-quality research, the intensity of future collaboration within and across regional spaces, and the emergence of continental science networks. Importantly, it encourages initiatives that aim at promoting collaborative science through the setting up of mixed top-down and bottom-up coordination combined with sound peer-based evaluation systems.

Finally, our study has some limitations that are worth noting. Although bibliometric studies can provide useful results and policy recommendations, the current analysis only involved an indirect assessment of the policy initiatives and frameworks that have been promoted both nationally and internationally in the region over time. As such, bibliometric-based measures concern only the production and dissemination of knowledge, grasping in this way only the output of the process. Therefore, this work cannot capture fully the geography of regional collaborations, especially if the domestic co-publications are issued in African journals that are not listed in Thomson Reuters' Web of Science.

## CRediT authorship contribution statement

**Mafini DOSSO**: Conceptualization (including policy framework); Formal analysis; Data analysis; Writing – review & editing, **Lorenzo CASSI**: Conceptualization; Data curation; Formal analysis; Writing – review & editing. **Wilfriedo MESCHEBA**: Data curation; Formal analysis; Networks visualizations.

## Declaration of competing interest

The authors whose names are listed immediately below certify that they have NO affiliations with or involvement in any organization or entity with any financial interest (such as honoraria; educational grants; participation in speakers' bureaus; membership, employment, consultancies, stock ownership, or other equity interest; and expert testimony or patent-licensing arrangements), or non-financial interest (such as personal or professional relationships, affiliations, knowledge or beliefs) in the subject matter or materials discussed in this manuscript.

## Figures and Tables

**Table C.1:** Publication indicators by REC and scientific discipline.

Disciplines	CEN SAD			COMESA		
	NB_PUB	% of domestic publications	Share of publications in Top 10 %	NB_PUB	% of domestic publications	Share of publications in Top 10 %
Applied Biology - Ecology	27,558	28 %	9 %	20,417	23 %	10 %
Chemistry	35,778	35 %	8 %	23,623	37 %	9 %
Computer Science	23,727	58 %	6 %	9664	53 %	9 %
Earth Sciences, Astronomy, Astrophysics	20,335	32 %	9 %	12,863	28 %	10 %
Engineering	48,552	48 %	9 %	25,231	45 %	11 %
Fundamental Biology	22,264	26 %	8 %	16,840	24 %	9 %
Humanities	2971	49 %	8 %	2127	36 %	9 %
Mathematics	6534	43 %	10 %	2548	31 %	14 %
Medical Research	50,751	37 %	8 %	42,550	31 %	9 %
Multidisciplinary	1187	37 %	6 %	968	30 %	9 %
Physics	18,937	34 %	12 %	10,602	34 %	14 %
Social Sciences	17,967	35 %	8 %	13,742	24 %	9 %
Total	276,561			181,175		


Disciplines	EAC			ECCAS		
	NB_PUB	% of domestic publications	Share of publications in Top 10 %	NB_PUB	% of domestic publications	Share of publications in Top 10 %
Applied Biology - Ecology	6464	11 %	12 %	2332	9 %	8 %
Chemistry	766	17 %	8 %	1040	11 %	6 %
Computer Science	745	37 %	6 %	258	24 %	11 %
Earth Sciences, Astronomy, Astrophysics	2918	11 %	12 %	1222	14 %	8 %
Engineering	1483	17 %	9 %	896	23 %	11 %
Fundamental Biology	4334	5 %	13 %	1917	6 %	10 %
Humanities	1046	24 %	13 %	238	34 %	6 %
Mathematics	144	15 %	8 %	386	38 %	16 %
Medical Research	13,717	9 %	11 %	4330	14 %	10 %
Multidisciplinary	336	12 %	12 %	92	14 %	11 %
Physics	314	14 %	13 %	770	39 %	10 %
Social Sciences	7349	13 %	11 %	1473	17 %	8 %
Total	39,616			14,954		


Disciplines	ECOWAS			IGAD		
	NB_PUB	% of domestic publications	Share of publications in Top 10 %	NB_PUB	% of domestic publications	Share of publications in Top 10 %
Applied Biology - Ecology	8059	25 %	7 %	7783	15 %	11 %
Chemistry	3840	24 %	7 %	1816	15 %	9 %
Computer Science	2008	43 %	5 %	1161	39 %	5 %
Earth Sciences, Astronomy, Astrophysics	5477	29 %	7 %	3889	13 %	11 %
Engineering	5970	35 %	7 %	2640	21 %	9 %
Fundamental Biology	5570	15 %	9 %	4739	9 %	11 %
Humanities	1351	54 %	6 %	946	24 %	12 %
Mathematics	881	34 %	12 %	250	19 %	8 %
Medical Research	16,565	28 %	9 %	15,697	20 %	10 %
Multidisciplinary	470	35 %	6 %	411	26 %	9 %
Physics	1715	23 %	13 %	776	13 %	13 %
Social Sciences	9293	36 %	7 %	8126	22 %	10 %
Total	61,199			48,234		


Disciplines	SADC			UMA		
	NB_PUB	% of domestic publications	Share of publications in Top 10 %	NB_PUB	% of domestic publications	Share of publications in Top 10 %
Applied Biology - Ecology	21,733	34 %	10 %	7999	33 %	7 %
Chemistry	11,735	44 %	9 %	16,598	35 %	7 %
Computer Science	6120	63 %	6 %	18,657	60 %	4 %
Earth Sciences, Astronomy, Astrophysics	17,164	31 %	12 %	8768	39 %	7 %
Engineering	16,910	55 %	8 %	33,402	53 %	6 %
Fundamental Biology	13,716	24 %	11 %	5269	28 %	6 %
Humanities	9112	60 %	9 %	601	54 %	6 %
Mathematics	3199	36 %	11 %	5366	51 %	7 %
Medical Research	33,649	25 %	11 %	8798	49 %	6 %
Multidisciplinary	1225	46 %	8 %	269	55 %	4 %
Physics	7902	30 %	17 %	12,162	40 %	8 %
Social Sciences	23,235	41 %	9 %	3275	50 %	7 %
Total	165,700			121,164		

**Table C.2:** Co-publication indicators by REC and scientific discipline.

Disciplines	CEN SAD				COMESA				EAC				ECCAS			
	% of co-publications with African countries outside the REC (in total co-publications)	% of co-publications with non-African countries (in total co-publications)	% of co-publications excl. between the countries of the REC (in total co-publications)	% of international co-publications involving at least another country of the REC	% of co-publications with African countries outside the REC (in total co-publications)	% of co-publications with non-African countries (in total co-publications)	% of co-publications excl. between the countries of the REC (in total co-publications)	% of international co-publications involving at least another country of the REC	% of co-publications with African countries outside the REC (in total co-publications)	% of co-publications with non-African countries (in total co-publications)	% of co-publications excl. between the countries of the REC (in total co-publications)	% of international co-publications involving at least another country of the REC	% of co-publications with African countries outside the REC (in total co-publications)	% of co-publications with non-African countries (in total co-publications)	% of co-publications excl. between the countries of the REC (in total co-publications)	% of international co-publications involving at least another country of the REC
Applied Biology - Ecology	4 %	94 %	1 %	8 %	5 %	94 %	1 %	7 %	8 %	90 %	2 %	10 %	7 %	92 %	1 %	8 %
Chemistry	4 %	96 %	1 %	3 %	2 %	98 %	0 %	1 %	15 %	84 %	1 %	4 %	8 %	91 %	0 %	2 %
Computer Science	2 %	98 %	1 %	2 %	3 %	97 %	0 %	1 %	11 %	88 %	2 %	4 %	8 %	91 %	1 %	2 %
Earth Sciences, Astronomy, Astrophysics	4 %	95 %	1 %	5 %	4 %	95 %	1 %	4 %	8 %	92 %	1 %	6 %	7 %	92 %	1 %	6 %
Engineering	4 %	96 %	1 %	2 %	3 %	97 %	0 %	2 %	13 %	86 %	1 %	5 %	12 %	88 %	1 %	2 %
Fundamental Biology	2 %	97 %	1 %	8 %	2 %	98 %	0 %	5 %	3 %	96 %	0 %	9 %	5 %	95 %	0 %	8 %
Humanities	7 %	92 %	1 %	6 %	9 %	91 %	1 %	5 %	6 %	93 %	1 %	6 %	4 %	93 %	3 %	6 %
Mathematics	4 %	95 %	1 %	2 %	4 %	96 %	1 %	1 %	19 %	80 %	1 %	3 %	23 %	76 %	1 %	1 %
Medical Research	3 %	96 %	1 %	9 %	2 %	97 %	0 %	7 %	3 %	97 %	0 %	9 %	8 %	92 %	0 %	7 %
Multidisciplinary	3 %	97 %	0 %	6 %	3 %	96 %	1 %	7 %	4 %	95 %	1 %	9 %	9 %	90 %	1 %	15 %
Physics	4 %	96 %	1 %	2 %	3 %	97 %	0 %	1 %	26 %	74 %	0 %	1 %	19 %	80 %	2 %	4 %
Social Sciences	5 %	93 %	1 %	9 %	5 %	95 %	1 %	8 %	5 %	94 %	1 %	7 %	7 %	93 %	0 %	5 %


Disciplines	ECOWAS				IGAD				SADC				UMA			
	% of co-publications with African countries outside the REC (in total co-publications)	% of co-publications with non-African countries (in total co-publications)	% of co-publications excl. between the countries of the REC (in total co-publications)	% of international co-publications involving at least another country of the REC	% of co-publications with African countries outside the REC (in total co-publications)	% of co-publications with non-African countries (in total co-publications)	% of co-publications excl. between the countries of the REC (in total co-publications)	% of international co-publications involving at least another country of the REC	% of co-publications with African countries outside the REC (in total co-publications)	% of co-publications with non-African countries (in total co-publications)	% of co-publications excl. between the countries of the REC (in total co-publications)	% of international co-publications involving at least another country of the REC	% of co-publications with African countries outside the REC (in total co-publications)	% of co-publications with non-African countries (in total co-publications)	% of co-publications excl. between the countries of the REC (in total co-publications)	% of international co-publications involving at least another country of the REC
Applied Biology - Ecology	9 %	89 %	2 %	13 %	8 %	91 %	1 %	8 %	6 %	91 %	3 %	10 %	1 %	97 %	2 %	6 %
Chemistry	22 %	78 %	1 %	2 %	11 %	89 %	0 %	2 %	13 %	85 %	2 %	5 %	2 %	97 %	1 %	4 %
Computer Science	8 %	91 %	1 %	3 %	11 %	89 %	0 %	1 %	7 %	88 %	4 %	6 %	1 %	99 %	1 %	2 %
Earth Sciences, Astronomy, Astrophysics	9 %	90 %	1 %	8 %	7 %	93 %	1 %	5 %	4 %	93 %	3 %	7 %	1 %	97 %	2 %	4 %
Engineering	16 %	83 %	1 %	4 %	10 %	90 %	0 %	4 %	11 %	85 %	4 %	6 %	1 %	98 %	1 %	2 %
Fundamental Biology	5 %	94 %	1 %	14 %	3 %	96 %	0 %	7 %	3 %	95 %	1 %	9 %	1 %	97 %	2 %	5 %
Humanities	13 %	86 %	1 %	7 %	6 %	94 %	1 %	6 %	3 %	93 %	3 %	6 %	1 %	99 %	0 %	3 %
Mathematics	16 %	83 %	1 %	2 %	19 %	81 %	0 %	1 %	7 %	90 %	3 %	4 %	1 %	97 %	1 %	3 %
Medical Research	6 %	93 %	1 %	13 %	3 %	97 %	0 %	7 %	4 %	95 %	1 %	11 %	2 %	96 %	1 %	6 %
Multidisciplinary	5 %	95 %	0 %	7 %	4 %	95 %	0 %	6 %	4 %	95 %	1 %	8 %	1 %	99 %	0 %	6 %
Physics	19 %	80 %	1 %	2 %	19 %	81 %	0 %	1 %	8 %	91 %	1 %	2 %	2 %	97 %	1 %	3 %
Social Sciences	8 %	90 %	2 %	10 %	5 %	95 %	1 %	6 %	5 %	93 %	2 %	9 %	2 %	98 %	1 %	4 %

**Table D.1:** Dyadic average characteristics of REC.

Region	Number of members (2019)	Number of dyads	Average geographical distance	Share of dyads having a colonial tie	Share of dyads belonging to CAMES	Share of dyads having same official language	Share of dyads having same ethnical language
Africa	53	1378	3657	0.26	0.12	0.44	0.32
Cen_sad	25	300	3079	0.42	0.18	0.49	0.34
Comesa	20	190	2996	0.28	0.03	0.56	0.41
Eac	5	10	685	0.40	0.10	0.70	0.50
Eccas	10	45	1335	0.27	0.62	0.64	0.42
Ecowas	15	105	1328	0.33	0.34	0.37	0.34
Igad	6	15	1272	0.40	0	0.60	0.20
Sadc	14	91	2091	0.41	0.01	0.56	0.38
Uma	5	10	1855	0.60	0	1	0.60

**Table D.2:** Correlation matrix.

	copub	logpub_i	logpub_j	geo_prox	logpas ~ b	comcol	scienc ~ x	lang_eth	logeu	cames	cen_sad	comesa	eac	eccas	ecowas	igad	sadc
copub	1.000																
logpub_i	0.262	1.000															
logpub_j	0.246	0.326	1.000														
geo_prox	0.112	−0.055	−0.018	1.000													
logpastcopub	0.588	0.491	0.490	0.252	1.000												
comcol	0.041	−0.012	0.030	0.123	0.160	1.000											
science_prox	0.150	0.194	0.288	0.135	0.344	0.027	1.000										
lang_eth	0.137	0.028	0.106	0.091	0.205	0.406	0.000	1.000									
logeu	0.336	0.508	0.529	0.147	0.803	0.129	0.333	0.150	1.000								
cames	−0.005	−0.104	−0.036	0.247	0.098	0.251	0.055	0.245	0.065	1.000							
cen_sad	−0.013	−0.001	0.054	0.166	0.050	0.174	0.078	0.006	0.043	0.072	1.000						
comesa	−0.009	0.036	0.021	0.130	0.003	0.002	0.060	0.080	0.013	−0.090	−0.093	1.000					
eac	0.150	0.052	0.030	0.186	0.152	0.036	0.057	0.032	0.088	−0.008	−0.039	0.039	1.000				
eccas	−0.027	−0.156	−0.073	0.268	−0.037	0.002	−0.059	0.045	−0.055	0.292	−0.084	−0.036	−0.015	1.000			
ecowas	0.033	−0.028	0.022	0.441	0.148	0.049	0.094	0.013	0.091	0.191	0.343	−0.101	−0.022	−0.054	1.000		
igad	0.053	−0.001	−0.007	0.161	0.047	0.043	0.034	−0.018	0.015	−0.042	0.017	0.122	0.079	−0.021	−0.032	1.000	
sadc	0.071	0.070	0.025	0.212	0.122	0.092	0.086	0.040	0.098	−0.092	−0.136	0.145	−0.020	−0.034	−0.076	−0.029	1.000
uma	0.068	0.045	0.067	0.085	0.088	0.067	0.015	0.051	0.059	−0.032	0.072	−0.027	−0.007	−0.016	−0.025	−0.010	−0.023

## Data Availability

The authors do not have permission to share data.

## Appendix A Regional Economic Communities and African Union regions

“Regional Economic Community” means a corporate legal entity established by its Treaty as defined in Article l(d) of the Treaty and whose objective is to promote economic integration was a step towards the establishment of the Community. (AEC protocol at https://wits.worldbank.org/GPTAD/PDF/annexes/AEC_protocols.pdf).

The African Union (AU) recognises 8 Regional Economic Communities (RECs, number of members, see also at: https://au.int/en/organs/recs, access Feb 2018):

• Arab Maghreb Union (AMU, 5): Algeria, Libya, Mauritania, Morocco, Tunisia
• Common Market for Eastern and Southern Africa (COMESA, 19): Burundi, Comoros, DR Congo, Djibouti, Egypt, Eritrea, Ethiopia, Kenya, Libya, Madagascar, Malawi, Mauritius, Rwanda, Seychelles, Sudan, Swaziland, Uganda, Zambia, Zimbabwe
• Community of Sahel-Saharan States (CEN-SAD, 29): Benin, Burkina Faso, Cabo Verde, Central African Republic, Chad, Comoros, Côte d'Ivoire, Djibouti, Egypt, Eritrea, Gambia, Ghana, Guinea, Guinea Bissau, Kenya, Liberia, Libya, Mali, Mauritania, Morocco, Niger, Nigeria, São Tomé and Príncipe, Senegal, Sierra Leone, Somalia, Sudan, Togo, Tunisia
• East African Community (EAC, 5): Burundi, Kenya, Rwanda, Uganda, UR of Tanzania
• Economic Community of Central African States (ECCAS, 10): Angola, Burundi, Cameroon, Central African Republic, Chad, Congo, DR Congo, Equatorial Guinea, Gabon, São Tomé and Príncipe
• Economic Community of West African States (ECOWAS, 15): Benin, Burkina Faso, Cabo Verde, Côte d'Ivoire, Gambia, Ghana, Guinea, Guinea Bissau, Liberia, Mali, Niger, Nigeria, Senegal, Sierra Leone, Togo
• Intergovernmental Authority on Development (IGAD): Djibouti, Eritrea (check status on website), Ethiopia, Kenya, Somalia, South Sudan, Sudan, Uganda
• Southern Africa Development Community (SADC, 15): Angola, Botswana, DR Congo, Lesotho, Madagascar, Malawi, Mauritius, Mozambique, Namibia, Seychelles, South Africa, Swaziland, UR of Tanzania, Zambia, Zimbabwe

## Appendix B Sources for the elaboration of Table 1

The main sources for the five columns of Table 1 include (access May 2021):

Columns (1) and (2) are mainly based on the AU's REC page: https://au.int/en/organs/recs and, the active and official REC websites as of May 2021 (see column 2).

Notes:

- AMU is not a signatory to the Protocol between the RECs and the AU
- COMESA: The Common Market envisaged in the Preferential Trade Agreement (1982) was created in 1993 under the COMESA Treaty that entered into force in 94 (Mangeni and Juma, 2019).
- EAC's historical details can be found at https://www.eac.int/eac-history
- CEN-SAD became a REC in 2000 (Gnanguênon, 2020). HQ were located in Libya before 2019.
- ECCAS Treaty was signed in October 1983 in Libreville, Gabon, and entered into force in December 1984. ECCAS ceased activities on 1992–1998. It was revitalized by a Heads of State and Government decision at the 1998 Summit in Libreville.
- ECOWAS revised Treaty can be found at https://www.ecowas.int/wp-content/uploads/2015/01/Revised-treaty.pdf
- IGAD is the successor to the Intergovernmental Authority on Drought and Development (IGADD), which was founded in 1986.
- SADC is the successor to the Southern African Development Co-ordination Conference (SADCC), which was established in 1980 in Lusaka, Zambia.

Column (3) is mainly based on official REC website (news or communique) combined with cross-checks through Google news search performed in May 2021.

Notes:

- AMU: see the AU's REC page and the discussion paper by Parshotam (2020)
- CEN-SAD: “ The organisation's most recent Conference of Heads of State and Government was held in February 2013 in Ndjamena, Chad.” (AU's REC page); see also one-day Extraordinary Session of the Conference of Heads of State and Government of the Community of Sahel-Saharan States (CEN-SAD).
- COMESA: https://www.comesa.int/wp-content/uploads/2019/02/Post-summit-2018_en_web.pdf and the news section: https://www.comesa.int/?s=summit
- EAC: see the press release at https://www.eac.int/communique/1942-communiqué-of-the-21st-ordinary-summit-of-the-east-african-community-heads-of-state
- ECCAS: press release available in French at https://gabon24.ga/actualite/gabon/communique-final-de-la-18eme-session-ordinaire-de-la-ceeac/1924/
- ECOWAS: The final communique can be found at https://www.ecowas.int/wp-content/uploads/2021/01/UK-Communiqué-Final-58th-AHSG_VF.pdf
- IGAD: The final communique is available at https://igad.int/communique/2465-communique-of-the-36th-extraordinary-assembly-of-igad-heads-of-state-and-government-held-online-via-videoconference-14th-july-2020-2
- SADC: See the news section at https://www.sadc.int/news-events/news/40th-sadc-ordinary-summit-heads-state-and-government-be-held-virtually-10-17th-august-2020/

Columns (4) and (5) are mainly based on the latest STISA-2024 implementation report (AU, 2019).

Additional searches were made on the official REC websites (when they were active).

Notes:

- EAC, see at https://easteco.org/wp-content/uploads/2020/06/EASTECO-Strategic-Plan-Jan-130427-Approved-by-35th-Council_4th-April-2017-1.pdf
- ECOWAS's ECOPOST and SADC 2008 protocol, see UNESCO (2015). For ECOWAS, see also a related 2012 directive at http://www.esc.comm.ecowas.int/wp-content/uploads/2016/04/ECOWAS-Directive-on-STI-Eng.pdf and for SADC see also https://www.sadc.int/themes/social-human-development/science-technology-innovation/
- IGAD 2016–2020 plan included STI as a priority area; see also a recent dedicated framework https://igad.int/attachments/article/2593/IGAD%20Regional%20Education%20Policy%20Framework%202020.pdf

## Footnotes

1 See Appendix A for the member countries. The protocol for the AEC–RECs relations can be found at https://wits.worldbank.org/GPTAD/PDF/annexes/AEC_protocols.pdf.

2 Other STI bodies were created under the AUC, including the African Science Research and Innovation Council, the Pan-African University, and the African Observatory of STI; see also the Treaties on Education, S&T at https://au.int/en/treaties/1167.

3 AESA is one of the four platforms of the African Academy of Sciences (AAS). Created in 1985, the AAS is a non-aligned, non-political, not-for-profit pan-African organisation, whose aim is to foster excellence and promote distinguished researchers (AAS fellows). Detailed information about their achievements and actions can be found at http://www.aasciences.africa/about.

4 Sixteen states initially adopted the convention creating the CAMES in Lomé in 1972. More information can be found at https://www.lecames.org/historique/.

5 The organisational proximity, which is not considered in the present analysis, reflects whether two or more actors belong to the same organisation or group. Actors working within the same organisation or group have stronger incentives and fewer costs for establishing a collaboration compared to actors that belong to different organisations. However, in our context, the organisation refers in the majority of cases to local or national universities and/or research centres rather than corporate multinational organisations, given the nature of the data (i.e. international science collaboration); hence, this dimension or proximity does not appear relevant for our analytical purposes and the nature of our data.

6 Thus, the total number of observations is given by (n × (n − 1) / 2) × *t* = 24,804, where *n* is the number of countries (i.e. 53) and *t* is the number of years, i.e. 18 years, from 2002 to 2019.

7 HCERES-OST: *Haut Conseil de l'évaluation de la recherche et de l'enseignement supérieur. Observatoire des Sciences et Techniques* (see https://www.hceres.fr/fr/observatoire-des-sciences-et-techniques-ost?%2F). One improvement is, for instance, the geo-localisation of authors.

8 A comprehensive comparison of WoS and Scopus (Mongeon and Paul-Hus, 2016) shows that Scopus is subject to the same biases as WoS, despite its larger journal coverage. This bias is hard to avoid for international comparative studies, and so should be kept in mind when exploiting the results.

9 Fundamental Biology, Medical Research, Applied Biology, Chemistry, Physics, Earth Sciences, Astronomy and Astrophysics, Engineering and Computer Sciences, and Mathematics, Social Sciences and Humanities, and Multidisciplinary. They are defined as an aggregation of Thomson Reuters' Scientific Categories.

10 The correlation coefficients can be found in the Statistical Appendix.

11 If the distance is equal to zero, then the proximity is assumed to be equal to one.

12 The correlation between common colonial heritage and official language was 0.57, while the correlation with ethnical language was 0.4.

13 See also Sooryamoorthy (2018), which exploits Web of Science data for an in-depth mapping of African publications at the country and field levels in the period 2000–2015.

14 The United States was the top-ranked non-African partner in the co-publications.

15 While the UK has not been a member of the EU since 2020, our period of analysis covers the years between 2002 and 2019 when it was still an EU member.

16 See Cissé (2018), for an in-depth historical discussion on CAMES for the period 1968–2018. Cames' current strategic plan is synthetised by the organisation at https://www.lecames.org/wp-content/uploads/2019/09/PLAN-Stratégique_Brochure_20-pages_VF.pdf.

17 The original strategy can be found at https://knowledge4policy.ec.europa.eu/publication/joint-africa-eu-strategy_en. The declaration is available at https://archives.au.int/handle/123456789/329 (Accessed in August 2022).
